# Peer-led medication education in the time of COVID-19

**DOI:** 10.1192/bjo.2021.429

**Published:** 2021-06-18

**Authors:** Rosa Roberts

**Affiliations:** Avon and Wiltshire Partnership Trust; Barnet Enfield and Haringey Mental Health NHS Trust

## Abstract

**Aims:**

To set up an online peer-led medical education programme for core psychiatry trainees during the COVID-19 pandemic.

To determine trainees’ views regarding the role of peer-led education in psychiatry.

**Method:**

A peer-led education programme was set up for psychiatry trainees in their third year of core training, held over an online video-conferencing platform. The weekly sessions were organised and led by trainees. Each week a trainee either presented a journal article or a particular psychiatric topic, with a group discussion afterwards.

An online survey was sent to psychiatry trainees that had participated in the programme to determine their views. Close-ended questions were asked as well as open-ended questions for more qualitative responses.

**Result:**

There were 9 peer-led sessions in total, with 11 trainees (out of 18 invited) attending at least one session, and an average of 5 trainees at each session.

Five core trainee psychiatrists responded to the survey following the sessions. All of the respondents found the sessions either “very” or “fairly” useful. 80% stated that there should be more peer teaching opportunities as part of normal psychiatry training. All respondents found engaging with online teaching either “easy” or “OK”.

Open-ended questions showed that respondents found the sessions were useful for: 1) connecting with peers during a difficult time 2) free discussion due to being around peers 3) wide interest and variety of topics. Improvements that could have been made were: 1) more sessions 2) advance knowledge of journal articles being presented.

**Conclusion:**

Peer-led sessions are a useful form of medical education for core psychiatry trainees. During the restrictions brought about by the COVID-19 pandemic they are a way to connect with colleagues during an isolating time. They are easy to organise and access; and can take pressure off medical institutions, whilst having the advantages of allowing trainees to feel they can discuss topics in a more open manner and can follow their own interests.

